# A single-arm feasibility study of community-delivered Baduanjin (Qigong practice of the eight Brocades) training for frail older adults

**DOI:** 10.1186/s40814-020-00649-3

**Published:** 2020-07-21

**Authors:** Xiao Liu, Jean Wei Ting Seah, Benedict Wei Jun Pang, Mary Ann Tsao, Falong Gu, Wai Chong Ng, Junie Ying Ru Tay, Tze Pin Ng, Shiou Liang Wee

**Affiliations:** 1 Geriatric Education and Research Institute, 2 Yishun Central 2, Tower E Level 4 GERI Admin, Singapore, 768024 Singapore; 2 Tsao Foundation, Singapore, Singapore; 3 Hua Mei Acupuncture and TCM Centre, Tsao Foundation, Singapore, Singapore; 4 Clinical Affairs, Tsao Foundation, Singapore, Singapore; 5 Kwong Wai Shiu Hospital, Singapore, Singapore; 6 Gerontology Research Programme, Department of Psychological Medicine, Yong Loo Lin School of Medicine, National University of Singapore, Singapore, Singapore; 7 Health and Social Sciences Cluster, Singapore Institute of Technology, Singapore, Singapore

**Keywords:** Baduanjin, Exercise program, Prefrail/frail, Community, Pilot study

## Abstract

**Background:**

Frailty is a common geriatric syndrome, characterized by reduced physiologic reserve and increased vulnerability to stressors, due to cumulative decline in multiple physiological systems. We studied the feasibility of a community-delivered Baduanjin (BDJ) training program among pre-frail/frail community-dwelling older people. We examined (1) safety (adverse events) and physical and psychological effects; and (2) feasibility of recruitment, retention, adherence; recruitment efforts, and any program challenges, so as to inform future studies.

**Methods:**

Our study was a single arm pre-post study in a community setting. Sixteen-week group BDJ training (2×/week in the first 4 weeks and 3×/week thereafter) was co-designed and implemented by community-based providers in Singapore. Recruitment, attendance, and adverse events were recorded throughout the training. A participants’ survey was also administered after the training program. Effects of the intervention on physical and functional outcomes (hand grip strength, knee extension strength, Time Up and Go (TUG), Physiological Profile Assessment (PPA), 30-s Sit-to-Stand test, 6-m fast gait speed test), frailty outcomes (frailty score and status), and other outcomes (Maastricht Questionnaire (MQ), Fall Efficacy Scale (FES), Montreal Cognitive Assessment (MoCA), Geriatric Depression Scale (GDS), and EQ-5D-5L) were examined before and after the program.

**Results:**

Of 31 older adults screened to be frail, 15 met inclusion criteria and 3 refused participation, resulting in 12 older adults (9 women) enrolled into the program. During the program, one participant was hospitalized (unrelated to BDJ training) and the other 11 (aged 77 ± 6 years; 2 frail, 9 prefrail at baseline) completed the program with average overall attendance of 89%. Most (89%) of the 44 training sessions had attendance > 80%. The program received positive feedback with no training-related adverse events. Participants either reversed (*n* = 2) or maintained (*n* = 9) their frailty statuses. There post-training outcomes in hand grip strength, knee extension strength, TUG, MQ, FES, MoCA, GDS, and EQ-5D-5L index score appeared to be better. The reduction of frailty and PPA fall risk scores was of moderate-to-large effect size.

**Conclusions:**

Community-delivered BDJ training program was safe and feasible for prefrail/frail older adults with the potential to improve physical and cognitive function, reduce fall risk, improve psychological well-being, and reverse frailty status.

## Key messages regarding feasibility

- What uncertainties existed regarding the feasibility?
  There is no information on the safety, acceptability, and adherence of community-delivered BDJ training among prefrail/frail older adults.
- What are the key feasibility findings?
  The BDJ training was safe with very good attendance and acceptance among the prefrail/frail community dwelling older adults.
- What are the implications of the feasibility findings for the design of the main study?
  The results of this feasibility study can inform the design and development of randomized controlled study in terms of protocol feasibility, sample size calculation, and outcome selection.

## Background

Frailty is a common geriatric syndrome, characterized by reduced physiologic reserve and increased vulnerability to stressors, due to cumulative decline in multiple physiological systems [1]. The prevalence of frailty among community-dwelling older people has been reported to be between 4.0 to 59.1% [2]. A quarter to a half of people older than 85 years has been estimated to be frail [3]. Recently, frailty has been recognized as a public health priority because of its high prevalence and predictive capacity for functional disability, hospitalization, institutionalization, and death [4, 5]. The frailty process is a transitional, with dynamic progression from robust to prefrail and to frail, which can improve or worsen over time [3, 6, 7]. This highlights the importance of frailty identification, prevention, reduction, and management.

Different interventions to reduce frailty have been studied [8–10]. Recent reviews highlighted the importance of physical exercise in reducing frailty and improving function in prefrail/frail older adults [11, 12]. Economic study also demonstrated that physical exercise is cost-effective in preventing the progression of frailty and disability in older adults living in the community [13]. However, compared with the general older population, prefrail/frail older adults have relatively lower tolerance to intense physical exercise and are usually more sedentary than non-frail counterparts (low physical activity being a defining feature of frailty) in the same age group. It is therefore important to consider the acceptability, tolerability, and safety in recommending physical training program for prefrail/frail older adults to participate and enjoy in the community where they lived.

Baduanjin (BDJ), also known as Eight-Section Brocades, is one common form of “mind-body” Qigong. “Mind” is understood as regulating thoughts to enhance the emotional and psychological processes. It involves eight simple movements with combinations of postures, meditation, slow relaxing movements, and breathing exercise in a harmonious manner which enhances respiratory function, improves holistic health, and achieves the integration of mind and body [14]. A growing number of BDJ demonstrations are available on social media, such as YouTube (https://www.youtube.com/watch?v=3K-0JpiJu-o&t=75s). Compared with relatively more complex and lengthy exercise forms such as Tai Chi, BDJ is physically and cognitively less demanding and is easy to learn and practice with few limitations [15]. Thus, it may be suitable for older adults and those who are frail. Systematic reviews [16, 17] have indicated that BDJ is popular and confers health benefits including physical fitness [18–20], mental health [21, 22], and quality of life [23–25] in different populations. Among those showing improvement in quality of life, the frequency of BDJ varied widely from daily to monthly [23, 26]. However, to our knowledge, there has yet been any study on BDJ training in frail older adults.

There are many factors to consider in translating the result of a successful clinical trial into real life settings [27]. As such, there is merit in implementing an intervention in a community setting where it is most applicable. Furthermore, considering the vulnerabilities of the target population of frail older adults, there is a need for a feasibility study to first understand the safety, acceptance, and adherence of the intervention to be delivered in a community setting. The primary aim of this single arm study was to determine the safety, acceptability of, and adherence to community-delivered BDJ program among pre-frail and frail older adults. We also aimed to estimate the effect sizes of the potential functional outcomes in order to inform sample size estimation of a randomized controlled study in the same community-dwelling population.

## Methods

### Setting and participants

This feasibility study was designed as a single arm pre-post comparison. In partnership with a provider of senior community services (day care and community clinic), the study was implemented at a community center from September 2018 to April 2019, where the provider is based at the Whampoa housing estate in Singapore. A small convenience sample of older adults was recruited by the provider among the visitors of the community center, daycare center, and community clinic at the same locale near enough for participants to walk to the training venue. Wheelchair users were either assisted by their domestic helper or used a paid transport we provided. Participants were familiar with the venues since those venues were the activity centers within the communities.

Researchers did initial frailty screening. Afterwards a licensed General Practitioner (GP) screened for program eligibility and excluded participants who did not meet the medical criteria. Since there was no previous study on BDJ frail older adults, we could not estimate sample size. Based on a maximum instructor to participant ratio of 1:10, we aimed to recruit a maximum of 20 participants.

The inclusion criteria were (1) pre-frail (score 1–2) or frail (scored 3–5) according to FRAIL scale screening questionnaire [28]; (2) older adults aged 55 years and above; (3) able to ambulate without personal assistance and has no other physical limitations which limited participation; (4) able to understand basic instructions; and (5) generally sedentary lifestyle according to Physical Activity Scale for the Elderly (PASE) questionnaire [29] (self-reported participation in sitting activities at least 5 days per week with more than 4 h per day on average).

Exclusion criteria were as follows: (1) participating in any other exercise program or interventional studies; (2) severe audio-visual impairment diagnosed by physicians (e.g., dementia, Alzheimer’s disease); (3) diagnosed with cognitive impairment and/or history of neurological disorder (e.g., cerebral palsy, Parkinson’s disease); (4) unable to participate for the full duration of the study; (5) diagnosed with postural hypotension; (6) unable to come to community club by himself/herself or by the help of caregivers, (7) unable to get the medical clearance from the GP.

### BDJ training program

**Fig. 1:**
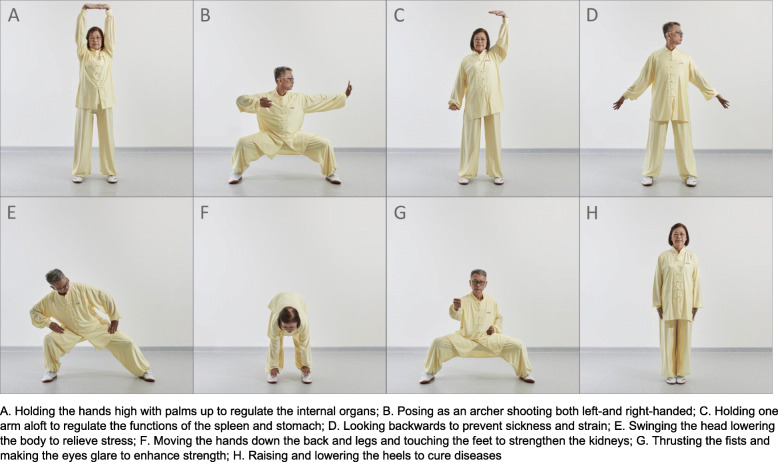
Baduanjin exercise

The program was designed by the local Qigong association. The entire set of BDJ exercise comprises eight sections (Fig. 1). Participants underwent a group-based 16-week supervised BDJ training program with a total of 44 training sessions. Each session lasted 90 min including warming up, BDJ exercise, and cool down. During the first 4 weeks, participants practiced each section of BDJ twice a week with the training intensity gradually increased to familiarize with the training. During the following 12-week period, participants practiced the whole set of BDJ exercise for 90 min three times a week. Two professional Qigong trainers from the local Qigong association conducted the training in both English and Chinese as appropriate.

All participants cleared pre-exercise medical examination before recruitment into the study. Simple screening was performed by study coordinator before each training session. Participant with either one of the following did not participate for that training session: blood oxygen levels below 95% saturation, high resting heart rate (≥ 90 beats per minute) [30], abnormal resting blood pressure (systolic blood pressure ≥ 130 mmHg or diastolic blood pressure ≥ 80 mmHg) [31], giddiness, or any form of discomfort. During training, each participant had a chair with armrests behind them so anyone who felt unstable can sit down. Two community volunteers assisted to ensure the seniors’ safety during exercise.

### Measurements

#### Program feasibility

To examine the feasibility of the training program, the outcomes and process evaluated were [32]:

1. Safety (adverse events) and physical and psychological effects.
2. Feasibility of recruitment, retention, adherence; recruitment efforts and any program challenges.
  A survey was administered to the participants after the training program. The survey utilized a four-point Likert scale (1 = strongly disagree, 2 = disagree, 3 = agree, and 4 = strongly agree) and comprised nine questions on program acceptance, adherence satisfaction, engagement, and willingness for continued participation. Any adverse event during the program duration was recorded by a research coordinator who contacted the participants regularly. The coordinator also obtained the attendance record from the provider and is present at every training session.

#### Physical and psychological outcomes

To explore the potential effects of BDJ, we assessed a range of exploratory outcomes during the week before (pre) and the week after (post) the 16-week training program. All outcome measures were administered by assessors who were not involved as part of the research team nor BDJ instruction and demonstration.

#### Physical and functional outcomes

Physical and functional outcomes included knee extension strength, hand grip strength, 6-m fast gait speed test, Time Up and Go test (TUG), Physiological Profile Assessment (PPA), and 30-second Sit-to-Stand (30 s STS) test.

To assess muscle strength, knee extension strength and hand grip strength were measured using a spring gage and hand-grip dynamometer respectively for the dominant leg or hand. Two trials were administered for knee extension strength, hand grip strength, and 6-m fast gait speed test and the mean values used for analyses.

TUG, a test of basic functional mobility for frail elderly persons, required participants to stand up from a chair, walk a distance of 3 m at a comfortable pace, turn, walk back, and sit down [33]. The test was performed twice, with the shorter time of two trials used for analysis.

PPA, which includes five measures of physiological functioning (postural sway, knee extension strength, reaction time, lower limb proprioception, and visual contrast sensitivity), was used as a systematic approach to explore the underlying causes of balance changes [34]. The five components were weighted to compute a composite PPA fall risk score, where higher composite scores indicate higher risks of falling.

For the 30 s STS, the number of successful stands within 30 s were recorded. The performance of 30 s STS has been suggested to be determined by balance, muscle strength, lower extremity endurance, and mobility [35].

#### Frailty outcomes

Frailty score of the participants was assessed using the Cardiovascular Health Study Frailty Phenotype (CHS Fried criteria) [1]. The following five criteria were: (1) shrinking (defined as unintentional weight loss > 4.5 kg and/or BMI of < 18.5 kg/m2 in the last 6 months by self-report); (2) weakness, assessed by knee extension strength of the dominant leg according to cut off points stratified by gender and BMI (Asian classification) ((a) in men: underweight < 9.5 kg, normal weight < 12.3 kg, overweight < 14.7 kg, obese < 15.0 kg; (b) in women: underweight < 9.3 kg, normal weight < 10.0 kg, overweight < 10.0 kg, obese < 10.0 kg); (3) slowness, established according to a cutoff point of < 0.8 m/s by 6-meter fast gait speed test; (4) exhaustion was measured with the 3 questions on vitality domain in the Medical Outcomes Study 12-Item Short-Form Health Survey [36] “Did you feel worn out?” “Did you feel tired?” “Did you have a lot of energy?” with total summed scores ranging from 3 to 15, and a score of less than 10 was used to denote exhaustion; and (5) physical inactivity, measured by Longitudinal Aging Study Amsterdam Physical Activity Questionnaire [37]. One-point was assigned for the presence of each component, and based on the individual’s total score, participants were categorized as frail (3–5 points), pre-frail (1–2 points), and robust (0 point). Reduction in frailty during the study was defined as a transition to a lower frailty category from baseline to post-training program.

#### Psychological and other outcomes

We also measured participants’ vital exhaustion using Maastricht Questionnaire (MQ) [38] and fear of falling using Fall Efficacy Scale (FES) [39]. Other outcomes also included Montreal Cognitive Assessment (MoCA) [40] for cognitive function, EQ-5D-5L [41] index score for the health-related quality of life, and the 16-item short form Geriatric Depression Scale (GDS) [42] to assess depression status.

### Statistical analyses

Descriptive statistics of demographic information and outcome measurements were presented as mean and standard deviation (SD) for continuous variables, and frequencies and percentages for categorical variables. Results from the outcome measurements were summarized, and effect size of each test was calculated using Cohen’s *d*. Results of the post intervention interview was reported as the percentage of responses for each question. All analyses were conducted using Stata 14.0 (StataCorp LP, College Station, TX).

## Results

### Characteristics of the participants

During the 3-month screening and recruitment in the community, 31 prefrail/frail older adults were identified using FRAIL scale. Of those, 16 who not meet inclusion criteria were excluded, and another three declined participation. In the end, twelve participants aged between 65 to 84 years old (mean = 77.1, SD = 5.9) were enrolled at baseline. The participants were all Chinese and had an average of 2.75 chronic illnesses—hypertension (91.7%), diabetes (50%), hyperlipidemia (41.7%), arthritis (33.3%), and heart disease (25%) were the top five comorbidities. Fifty percent of the participants used walking aids. According to CHS Fried criteria, 10 participants were prefrail, and two were frail (Table 1).

**Table 1:** Baseline characteristics of study participants (*n* = 12)

Demographics	Mean (SD) or frequency (%)
Age	77.1 (5.9)^a^
Gender	
Female	9 (75)^b^
Male	3 (25)
Race/Ethnicity	
Chinese	12 (100)
Education	
No formal education	3 (25)
Primary school	2 (16.7)
Secondary school	7 (58.3)
Living status	
With family	11 (91.7)
Alone	1 (8.3)
Marital status	
Married	11 (91.7)
Single	1 (8.3)
Smoking status	
Non-smoker	11 (91.7)
Ex-smoker	1 (8.3)
Alcohol intake	
Non-alcohol	11 (91.7)
Regular	1 (8.3)
Falls in past 6 month	4 (33.3)
Frailty Status (Fried criteria)	
Prefrail	10 (83.3)
Frail	2 (16.7)
BMI	26 (3.7)
Comorbidities	2.8 (2)
Hypertension	11 (91.7)
Diabetes	6 (50)
Hyperlipidemia	5 (41.7)
Arthritis	4 (33.3)
Heart disease	3 (25)
Mobility aids	6 (50)
Independent	6 (50)
Canes	3 (25)
Mobility walker	1 (8.3)
Wheelchair	2 (16.7)

*BMI* body mass index

^a^Data are presented as mean (standard deviation) for all such values

^b^Data are presented as frequency (%) for all such values

### Feasibility of BDJ training program

**Fig. 2:**
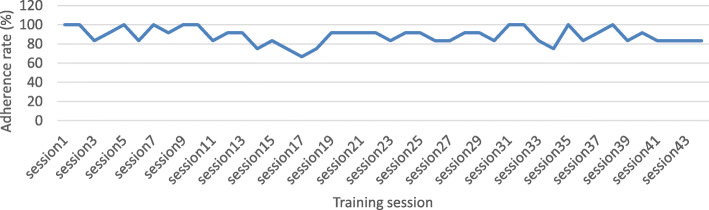
Adherence rate for each session of the program

Our study showed it was feasible to recruit sufficient prefrail/frail participants within a 3-month period. Among 12 participants, 11 completed the 16-week program, and one dropped out due to hospitalization unrelated to training program which showed a high retention rate of 91.7%. Overall, the group demonstrated good adherence, completing an average of 88.6% attendance for the 44 training sessions. Among the 44 sessions, 23% of the training sessions (*n* = 10) had full attendance, 66% (*n* = 29) had an attendance of 80–100%, and only 11% (*n* = 5) had an attendance of lower than 80% (Fig. 2).

**Fig. 3:**
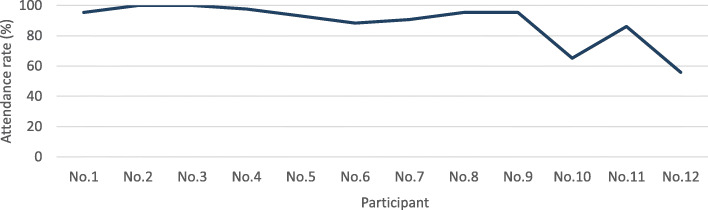
Attendance rate for each participant

Nine (83%) participants had an adherence of more than 80% throughout the training program (Fig. 3), among them, two participants achieved 100% program attendance. Of note, the two participants with lower adherence experienced one hospitalization episode each due to issues unrelated to the training.

All training and evaluation sessions were completed within the planned time period and within budget. Study did not record significant any personnel challenges or problems with respect to data collection.

Other than the single hospitalization unrelated to training, there was no other adverse event. Therefore, training program was found to be safe for these frail participants. Over the 16-week BDJ training program, there were no training-related fall, injuries, or serious adverse events.

**Fig. 4:**
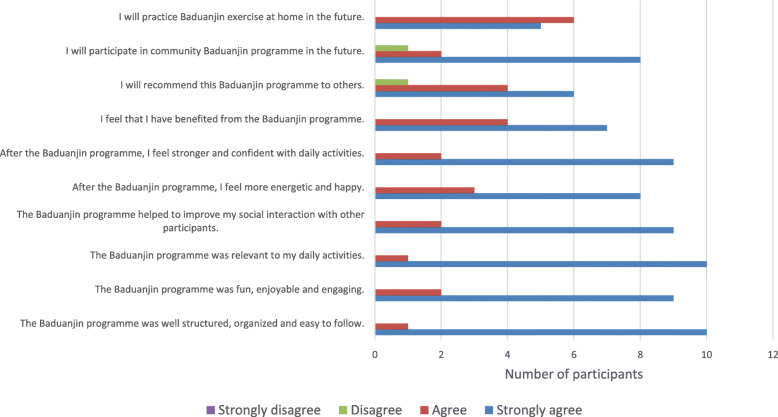
Responses from feedback survey (*n* = 11)

In addition, participants’ experience of the program was uniformly positive. As shown in Fig. 4, participants perceived the program to be engaging, with relevant physical, psychological, and social benefit. They would also recommend the program to others and continue the training after the program. Only two questions received one “Disagree” response each to the statements: “I will recommend this BDJ program to others” and “I will participate in such BDJ program in the future”, while all the rest indicated of “Agree” or “Strongly agree”.

### Potential effects of BDJ training program

Results of the 11 participants who completed the post assessments were analyzed. Pre-post changes in physical outcomes were apparent in TUG and hand grip strength, and knee extension strength had moderate-to-large effect size. A moderate effect size difference was shown in the reduction of PPA fall risk and frailty scores. Among the five components of PPA, better outcome was apparent in knee extension strength while other components appeared to be unchanged. There was no apparent change in the 6-meter fast gait and 30s STS tests (Table 2).

**Table 2:** Results of pre-post changes in physical outcomes for the 11 participants

Outcomes	Baseline (mean ± SD)	Post training (mean ± SD)	Mean of difference (Post vs baseline)			Cohen’s d effect size	Participants achieved better outcomes *n* (%)
			Mean ± SD	95% CI	%		
Time Up and Go (s)	16.1 ± 5.2	13.8 ± 4.4	− 2.3 ± 2.9	[− 4.4, − 0.2]	14.3	− 0.47	7 (64)
Grip strength (kg)	16.8 ± 5.5	18.4 ± 5.8	1.6 ± 2.0	[0.2, 2.9]	9.5	0.28	8 (73)
Knee extension strength (kg)	14.5 ± 4.1	17.5 ± 4.3	2.9 ± 5.3	[− 0.6, 6.5]	20.2	0.69	6 (55)
PPA composite fall risk score	2.2 ± 1.4	1.5 ± 0.9	− 0.7 ± 1.4	[− 1.6, 0.3]	30.5	− .59	6 (55)
6-m fast gait speed (s)	1.2 ± 0.4	1.2 ± 0.4	0.0 ± 0.2	[− 0.2, 0.1]	1.0	− 0.06	3 (27)
30-s Sit-to-Stand test	12.3 ± 3.0	11.5 ± 3.7	− 0.7 ± 2.6	[− 2.5, 1.0]	5.9	− 0.22	5 (45)
Frailty score (Fried criteria)	1.5 ± 0.8	1.2 ± 0.4	− 0.4 ± 0.7	[− 0.8, 0.1]	24.0	− 0.56	3 (27)
Fall Efficacy Scale	28.8 ± 9.4	23.3 ± 6.2	− 5.4 ± 7.9	[− 10.8, − 0.2]	18.8	− 0.68	7 (64)
MQ score	15.0 ± 8.1	8.6 ± 5.6	− 6.6 ± 5.1	[− 9.8, − 2.9]	44.2	− 0.91	10 (91)
MoCA	22.5 ± 4.1	24.3 ± 4.8	1.8 ± 2.4	[0.2, 3.4]	8.1	0.41	9 (82)
GDS score	3.7 ± 3.5	2.2 ± 1.6	− 1.6 ± 2.4	[− 3.1, 0.1]	41.5	− 0.57	8 (73)
EQ-5D-5L Index score	0.7 ± 0.3	0.9 ± 0.2	0.2 ± 0.3	[0.0, 0.3]	21.9	0.66	5 (45)

By the end of the training program, 3 participants recorded reduced frailty scores with a within-person change of 24%. Both the frail participants (*n* = 2) reversed to prefrail status (Table 2).

There was an apparent trend towards better subjective outcomes. Among the 11 participants, post-participation MQ scores were higher in 10 (91%), MoCA in 9 (82%), GDS in 8 (73%), and FES in 7 (64%), respectively with a within-person change ranging from 8.1–44.2% (Table 2). Four participants who were categorized as depressive according to GDS at baseline reverted to normal.

## Discussion

With a rapidly aging population around the world, frailty has become a major public health concern for both the older adults and society [43]. As such, there is a need to provide evidence-based interventions targeting prefrail/frail older adults for community translation. The results of our feasibility study suggested that the 16-week community-delivered BDJ training program was safe with very good acceptance and adherence in the selected group and potentially effective in improving health, function, and psychological outcomes among prefrail/frail older adults.

We were able to recruit 31 participants and completed screening within the planned 3-month period. Singapore comprised of majority (78%) of ethnic Chinese. We did not manage to recruit other minority races—Malays or Indians. Program retention rate (91.7%) was high. We observed that the community provider arranged for snacks and drinks after each session, and the participants could socialize with the instructors and with each other after the sessions. There was also very good rapport between the instructors and participants which appeared to improve over the program. The social component [11] and ability of instructors to engage participants during and outside training were important. Since our participants were prefrail/frail older adults, they were at increased risk of hospitalization. The single hospitalization episode that was unrelated to the training occurred during the program, and the participant did not complete the program. Safety measures during the training were adequate.

Program adherence is a significant challenge for exercise interventions in frail persons [44]. Poor program adherence leads to sub-optimal intervention dosage and health benefits. Prefrail/frail older adults with physical and/or cognitive function deficit may have greater difficulties adhering to exercise training. However, the feasibility of this community-delivered BDJ program is supported by the high average adherence rate of 88.6%, as well as result from participants’ survey. Even if our pre-defined 44-session was a little intense, this high adherence rate implied that the dose of intervention was still acceptable among prefrail/frail elderlies, and it could be the reference dose for our follow-up randomized clinical trial (RCT) design. It was noted that those participants even performed BDJ at home during the 16-week intervention as well as spontaneously continued with program in the community after the completion of the study.

A systematic review showed that the older people’s adherence to exercise programs was associated with the program characteristics and personal factors [45]. It was also suggested that the simple and slow movement nature of BDJ made it a suitable option for older adults [46]. The eight simple movements may enhance the older adults’ confidence in performing the exercise and compliance to training. Cultural identity and self-efficacy are important personal factors for adherence. Some older Chinese adults maybe keen to participate to BDJ due to favorable perceptions on Chinese Traditional Medicine (TCM). Moreover, to motivate participants’ participation, the Qigong trainers employed strategies to enhance the program experience—instructing the BDJ exercise in a fun way and nominating peer models in the class, which may have promoted self-efficacy, and greater enjoyment of the program participation. The post-training snacks and drinks further encouraged social interactions that further promoted participation, as supported by the program adherence and participant survey results. This underlines the importance of community partnership and other implementation factors in community program translation [47].

Falls have significant impacts on older adults including loss of mobility and confidence to maintain daily activities, leading to frequent hospitalizations and greater need for social care [48, 49]. Muscle strength and balance are two essential components of physical fitness which provide information about the older adults’ capacities to reduce fall risk. Furthermore, reduced strength and balance also restrict their performance of activities of daily living, especially among the prefrail/frail. Our results suggest that BDJ training may confer benefits to muscle strength and balance, with potential to reduce fall risk.

All training and evaluation sessions were completed within the planned time period and within budget. Study did not record significant any personnel challenges or problems with respect to data collection.

Program safety is vital for health promotion in the vulnerable population of frail older people. Our study intervention assessed BDJ exercise safety with close monitoring on blood pressure and blood oxygen levels before and after each training session. With no training-related adverse events, the current BDJ training protocol is safe for prefrail/frail older adults. The study exclusion criteria were adequate to ensure the safety for future trial. While intensive, the 44 training sessions within 4 months could maintain high adherence and retention rate. This showed that the BDJ training dose was acceptable in prefrail/frail older adults. In addition, the dose should be adequate to observe the physical and functional effectivity.

The participants appeared to have better post-training hand grip strength, lower limb strength, decrements in TUG and FES, better cognitive function (MoCA), psychological well-being (GDS), and quality of life (EQ-5D-5L index score). Frailty scores resulted in a marginal significant reduction of medium effect size (Cohen’s *d* = − 0.59). There may be possible detection bias since the assessors could not be blinded. However, for future RCT design, assessors should be blinded to the group allocation.

Two frail participants at baseline converted to prefrail, suggesting that BDJ could be a potential exercise for frailty reversal. It is worth noting that both of the frail participants were wheelchair users. This may indicate that older adults with lower functional capacity are more likely to benefit from slow movement exercise such as BDJ. Because participants could perform BDJ standing or seated, wheelchair users can also participate.

This is in accordance with the TCM theory that BDJ typically involves a mind–body integration to cultivate Qi (vital energy in TCM theory) for maximizing both physical and mental well-being [50]. Moreover, our study also used MQ, a validated tool, to explore the effects of Qi. The lower post-training MQ score may indicate the better outcome in vital energy after BDJ training. Future studies can employ the use of biochemical energetics to confirm the training effect of BDJ on Qi.

This study was the first to demonstrate that a community-based BDJ intervention is safe, feasible, and acceptable among prefrail/frail older persons. The high adherence rate is important for the implementation of such a program in the real-world setting. The strength of the study is the implementation in a “real-world” housing site setting where the participants reside, close partnership with local community providers to engage participation and adherence and using simple equipment such as chairs. To explore the potential effects of BDJ, we assessed a broad range of explorative outcomes including physical and cognitive functions as well as psychological outcomes. As fall risk is an important concern for prefrail/frail persons, both subjective (FES) and objective assessments (TUG and PPA) were conducted to assess fall risk from different perspectives. Furthermore, we explored the Qi component of BDJ exercise using the validated MQ instrument. The results of these explorative outcomes provide information to design a RCT on the effectiveness of BDJ to reduce frailty and improve function. Based on an effect size of 0.7 for within-subject in knee extension strength, with a power of 90% and a 10% drop-out rate, the sample size for a two-arm RCT will be 60 participants.

As a single-arm feasibility study, we could not make any assumptions on the effectiveness of the BDJ training program. The community provider also did not manage to recruit Malay or Indian participants. Future study in Singapore can include Malay and Indian participants to increase the generalizability. Although not a subject of investigation in this study, it is possible that BDJ Qigong is more appealing to the Chinese than Malay and Indians in Singapore due to its Chinese origin.

Our study highlights possible biases:

1. Detection bias: as our assessors could not be blinded in this single arm study. In a RCT, assessors should be blinded to reduce detection bias.
2. Performance bias: since our study was a physical intervention and participants could not be blinded in this nor a future RCT.
3. Attrition bias: while attrition was low in this study, attrition may be higher in a control group of a RCT design.

The results provide some support to BDJ’s potential to be implemented as part of low-cost community health promotion program. As BDJ is an exercise which is easy-to-learn without any restrictions from specialized equipment or spaces, older adults can practice it at home, community activity centers, or nursing homes, through both group-based and self-practiced training. Furthermore, BDJ is a traditional Chinese exercise which is popular among Asian Chinese populations. However, to translate the research intervention into a real-world routine community program, efforts from service providers and community partners who have a good intervention fidelity are necessary for a successful implementation. More strategies are needed to maximize the program adherence and achieve the desirable outcomes.

## Conclusion

In summary, the study provides early evidence that the 16-week community-delivered BDJ intervention adapted to prefrail/frail older adults is feasible, safe, and acceptable. It may potentially benefit prefrail/frail older adults on physical, cognitive, and psychological health outcomes, and frailty reduction and status reversal. The findings provided information for the design and implementation of a randomized controlled study to investigate the efficacy of BDJ exercise among prefrail/frail older adults.

## Data Availability

The data are de-identified participant data, which are available on request from the corresponding author Shiou Liang Wee (email: weeshiouliang@gmail.com), upon reasonable request. The data availability must also meet the ethical restrictions. No other additional information is available.

## Abbreviations

BDJ: Baduanjin
BMI: Body mass index
CI: Confidence interval
CHS Fried criteria: Cardiovascular Health Study Frailty Phenotype
FES: Fall Efficacy Scale
GDS: Geriatric Depression Scale
MoCA: Montreal Cognitive Assessment
MQ: Maastricht Questionnaire
PPA: Physiological Profile Assessment
RCT: Randomized control trial
SD: Standard deviation
TCM: Traditional Chinese Medicine
TUG: Time Up and Go

## References

[1] Fried LP, Tangen CM, Walston J, Newman AB, Hirsch C, Gottdiener J (2001). Frailty in older adults: evidence for a phenotype. J Gerontol Ser A Biol Med Sci.

[2] Collard RM, Boter H, Schoevers RA, Oude Voshaar RC (2012). Prevalence of frailty in community-dwelling older persons: a systematic review. J Am Geriatr Soc.

[3] Clegg A, Young J, Iliffe S, Rikkert MO, Rockwood K (2013). Frailty in elderly people. Lancet.

[4] Sloane PD, Cesari M (2018). Research on frailty: continued progress, continued challenges. J Am Med Dir Assoc.

[5] Cesari M, Prince M, Thiyagarajan JA, De Carvalho IA, Bernabei R, Chan P (2016). Frailty: an emerging public health priority. J Am Med Dir Assoc.

[6] Lang P-O, Michel J-P, Zekry D (2009). Frailty syndrome: a transitional state in a dynamic process. Gerontology..

[7] Morley JE, Vellas B, Van Kan GA, Anker SD, Bauer JM, Bernabei R (2013). Frailty consensus: a call to action. J Am Med Dir Assoc.

[8] Apóstolo J, Cooke R, Bobrowicz-Campos E, Santana S, Marcucci M, Cano A (2018). Effectiveness of interventions to prevent pre-frailty and frailty progression in older adults: a systematic review. JBI Database System Rev Implement Rep.

[9] Jadczak AD, Luscombe-Marsh N, Taylor P, Barnard R, Makwana N, Visvanathan R (2018). The EXPRESS study: exercise and protein effectiveness supplementation study supporting autonomy in community dwelling frail older people-study protocol for a randomized controlled pilot and feasibility study. Pilot and feasibility studies.

[10] GCV G, JMR B, do Socorro Simões M, Lin SM, LAP V, Varise EM (2017). Feasibility, safety, acceptability, and functional outcomes of playing Nintendo Wii Fit Plus™ for frail elderly: study protocol for a feasibility trial. Pilot and feasibility studies.

[11] Liu X, Ng DH-M, Seah JW-T, Munro YL, Wee S-L. Update on interventions to prevent or reduce frailty in community-dwelling older adults: a scoping review and community translation. Current Geriatrics Reports. 2019:1–15.

[12] Puts MTE, Toubasi S, Andrew MK, Ashe MC, Ploeg J, Atkinson E (2017). Interventions to prevent or reduce the level of frailty in community-dwelling older adults: a scoping review of the literature and international policies. Age Ageing.

[13] Yamada M, Arai H, Sonoda T, Aoyama T (2012). Community-based exercise program is cost-effective by preventing care and disability in Japanese frail older adults. J Am Med Dir Assoc.

[14] Zheng G, Chen B, Fang Q, Yi H, Lin Q, Chen L (2014). Primary prevention for risk factors of ischemic stroke with Baduanjin exercise intervention in the community elder population: study protocol for a randomized controlled trial. Trials.

[15] Zou L, Wang C (2017). Traditional Chinese Baduanjin Qigong for older adults: a mini-review. nursing.

[16] Zou L, SasaKi JE, Wang H, Xiao Z, Fang Q, Zhang M. A systematic review and meta-analysis of Baduanjin Qigong for health benefits: randomized controlled trials. Evid Based Complement Alternat Med. 2017;2017. 10.1155/2017/4548706 PMC5359459 28367223

[17] Guo Y, Shi H, Yu D, Qiu P (2016). Health benefits of traditional Chinese sports and physical activity for older adults: a systematic review of evidence. J Sport Health Sci.

[18] Xiao CM, Zhuang YC (2016). Effect of health Baduanjin Qigong for mild to moderate Parkinson’s disease. Geriatr Gerontol Int.

[19] Yun Liu X, Gao J, Xiang Yin B, Yu Yang X, Xi Bai D (2016). Efficacy of Ba Duan Jin in improving balance: a study in Chinese community-dwelling older adults. J Gerontol Nurs.

[20] Li M, Fang Q, Li J, Zheng X, Tao J, Yan X, et al. The effect of Chinese traditional exercise-Baduanjin on physical and psychological well-being of college students: a randomized controlled trial. PLoS One. 2015;10(7):e0130544-e. 10.1371/journal.pone.0130544 PMC4497728 26158769

[21] Chan JS, Li A, Ng S-M, Ho RT, Xu A, Yao T-J (2017). Adiponectin potentially contributes to the antidepressive effects of Baduanjin Qigong exercise in women with chronic fatigue syndrome-like illness. Cell Transplant.

[22] Zhang H, Zhu M, Song Y, Kong M (2014). Baduanjin exercise improved premenstrual syndrome symptoms in Macau women. J Tradit Chin Med.

[23] Hu GX, Gu KP (2014). Effects of Qigong eight section brocade exercise on quality of life of the elderly. Medicine and Society.

[24] Yu W. The influence of “Health Qigong Ba Duan Jin” training on the psychological health of college students [J]. Journal of Beijing Sport University. 2011;12.

[25] Liu X, Gao J, Zhang Q (2014). Influence of Ba Duan Jin exercise on quality of life of elderly in community. Journal of Nursing Care.

[26] Xiu MN (2015). Study on influence of Baduanjin exercise on cancer chemotherapy patients with cancer-related fatigue. Chinese General Practice Nursing.

[27] Tabak RG, Sinclair KA, Baumann AA, Racette SB, Sebert Kuhlmann A, Johnson-Jennings MD (2015). A review of diabetes prevention program translations: use of cultural adaptation and implementation research. Transl Behav Med.

[28] Morley JE, Malmstrom TK, Miller DK (2012). A simple frailty questionnaire (FRAIL) predicts outcomes in middle aged African Americans. J Nutr Health Aging.

[29] Logan SL, Gottlieb BH, Maitland SB, Meegan D, Spriet LL (2013). The Physical Activity Scale for the Elderly (PASE) questionnaire; does it predict physical health?. Int J Environ Res Public Health.

[30] Umetani K, Singer DH, McCraty R, Atkinson M (1998). Twenty-four hour time domain heart rate variability and heart rate: relations to age and gender over nine decades. J Am Coll Cardiol.

[31] Steffen TM, Hacker TA, Mollinger L (2002). Age-and gender-related test performance in community-dwelling elderly people: six-minute walk test, Berg Balance Scale, Timed Up & Go Test, and gait speeds. Phys Ther.

[32] Thabane L, Ma J, Chu R, Cheng J, Ismaila A, Rios LP (2010). A tutorial on pilot studies: the what, why and how. BMC Med Res Methodol.

[33] Podsiadlo D, Richardson S (1991). The timed “Up & Go”: a test of basic functional mobility for frail elderly persons. J Am Geriatr Soc.

[34] Lord SR, Menz HB, Tiedemann A (2003). A physiological profile approach to falls risk assessment and prevention. Phys Ther.

[35] Lord SR, Murray SM, Chapman K, Munro B, Tiedemann A (2002). Sit-to-stand performance depends on sensation, speed, balance, and psychological status in addition to strength in older people. J Gerontol Ser A Biol Med Sci.

[36] Ware JE Jr, Kosinski M, Keller SD. A 12-Item Short-Form Health Survey: construction of scales and preliminary tests of reliability and validity. Med Care. 1996:220–33. 10.1097/00005650-199603000-00003 8628042

[37] Stel VS, Smit JH, Pluijm SM, Visser M, Deeg DJ, Lips P (2004). Comparison of the LASA Physical Activity Questionnaire with a 7-day diary and pedometer. J Clin Epidemiol.

[38] Meesters C, Appels A (1996). An interview to measure vital exhaustion. II. Reliability and validity of the interview and correlations of vital exhaustion with personality characteristics. Psychol Health.

[39] Yardley L, Beyer N, Hauer K, Kempen G, Piot-Ziegler C, Todd C (2005). Development and initial validation of the Falls Efficacy Scale-International (FES-I). Age Ageing.

[40] Nasreddine ZS, Phillips NA, Bédirian V, Charbonneau S, Whitehead V, Collin I (2005). The Montreal Cognitive Assessment, MoCA: a brief screening tool for mild cognitive impairment. J Am Geriatr Soc.

[41] Herdman M, Gudex C, Lloyd A, Janssen M, Kind P, Parkin D (2011). Development and preliminary testing of the new five-level version of EQ-5D (EQ-5D-5L). Qual Life Res.

[42] Yesavage JA, Brink TL, Rose TL, Lum O, Huang V, Adey M (1982). Development and validation of a geriatric depression screening scale: a preliminary report. J Psychiatr Res.

[43] Buckinx F, Rolland Y, Reginster J-Y, Ricour C, Petermans J, Bruyère O (2015). Burden of frailty in the elderly population: perspectives for a public health challenge. Archives of Public Health.

[44] Woo J. Challenges of population ageing: putting frailty as a cornerstone of health and social care systems. Springer; 2018. 10.1007/s41999-018-0056-0 34654245

[45] Picorelli AMA, Pereira LSM, Pereira DS, Felício D, Sherrington C (2014). Adherence to exercise programs for older people is influenced by program characteristics and personal factors: a systematic review. J Phys.

[46] Zheng G, Fang Q, Chen B, Yi H, Lin Q, Chen L. Qualitative evaluation of baduanjin (Traditional Chinese Qigong) on health promotion among an elderly community population at risk for ischemic stroke. Evid Based Complement Alternat Med. 2015;2015. 10.1155/2015/893215 PMC4592925 26483845

[47] McKay HA, Sims-Gould J, Nettlefold L, Hoy CL, Bauman AE (2017). Implementing and evaluating an older adult physical activity model at scale: framework for action. Translational Journal of the American College of Sports Medicine.

[48] Leal J, Gray A, Prieto-Alhambra D, Arden NK, Cooper C, Javaid MK (2016). Impact of hip fracture on hospital care costs: a population-based study. Osteoporos Int.

[49] England PH. Falls: applying All Our Health [updated 24 July 2019. Available from: https://www.gov.uk/government/publications/falls-applying-all-our-health.

[50] Zou L, Pan Z, Yeung A, Talwar S, Wang C, Liu Y (2018). A review study on the beneficial effects of Baduanjin. J Altern Complement Med.

